# Home birth and its determinants among antenatal care-booked women in public hospitals in Wolayta Zone, southern Ethiopia

**DOI:** 10.1371/journal.pone.0203609

**Published:** 2018-09-07

**Authors:** Melese Siyoum, Ayalew Astatkie, Shewangizaw Mekonnen, Gezahegn Bekele, Kefyalew Taye, Zelalem Tenaw, Zemenu Yohannes, Zerai Kassaye

**Affiliations:** 1 School of Nursing and Midwifery, College of Medicine and Health Sciences, Hawassa University, Hawassa, Ethiopia; 2 School of Public and Environmental Health, College of Medicine and Health Sciences, Hawassa University, Hawassa, Ethiopia; 3 School of Medicine, College of Medicine and Health Sciences, Hawassa University, Hawassa, Ethiopia; Debre Markos University, ETHIOPIA

## Abstract

**Introduction:**

Antenatal care (ANC), health facility birth and postnatal care services are proved to reduce maternal and newborn morbidity and mortality. In Ethiopia, even though antenatal care coverage is good, still home birth is high. This study aimed to assess the prevalence and determinants of home birth among women who were booked for ANC in public hospitals in Wolaita zone, southern Ethiopia.

**Methods:**

A cohort study was conducted from February to May 2017 among 554 third trimester pregnant mothers who visited public hospitals of Wolaita Zone, southern Ethiopia for ANC service. All women were interviewed twice: the first interview was done face-to-face in the health facility in which they were having ANC follow up to gather information about basic socio-demographic and obstetric characteristics; the second interview was done via telephone after they gave birth to get information about the place of birth. Epi-Data version 3.1 was used for data entry and the Statistical Package for the Social Sciences (SPSS) version 22 was used for data analysis.

**Results:**

A total of 68 (13.5%; 95% Confidence Interval (CI): 10.5%-16.6%) women who were booked for ANC gave birth at home. Being uneducated (AOR = 2.46, 95% CI: [1.10–5.10]), starting ANC visit late (>16weeks) (AOR = 2.27, 95% CI: [1.14–4.50]), time taken to reach at health facility for ANC service **(>**30minutes) (AOR = 8.94, 95% CI: [4.50–17.72]), waiting time of greater than 30 minutes for ANC in health facilities (AOR = 1.18, 95% CI: [1.06–2.30]**)** and lack of knowledge about danger signs of pregnancy (AOR = 4.18, 95%CI: [1.80–9.70]) were significantly associated with home birth.

**Conclusions:**

Home birth among ANC booked women is low compared to other studies. Yet, giving attention to women with no education and those coming from far areas while providing advice on birth preparedness and pregnancy danger signs may be useful to further reduce the rate of home birth. Advising mothers to start ANC early and trying to reduce ANC waiting time could also be of importance.

## Introduction

Antenatal care (ANC) from a skilled provider is important to monitor pregnancy and reduce morbidity and mortality risks for the mother and the child during pregnancy, delivery, and the postnatal period (PNC)[1]. ANC comprises a set of interventions that a pregnant woman receives from organized health care services in order to prevent, identify and treat complications and help a woman approach pregnancy and birth as positive experiences[2]. It is proved that ANC, skilled attendance during delivery and PNC services have crucial role in reduction of perinatal mortality [3, 4]. However the prevalence of health facility birth in Ethiopia is still low, even though there is improvement over the last years. According to the Ethiopian Demographic Health Survey (EDHS) reports, the prevalence of health facility birth was 10% in 2011, 15.4% in 2014 and 26% in 2016. Despite this low coverage of health facility birth, the ANC coverage is increasing over time, especially ANC-1 visit is high[1, 5–7].

Studies have shown mothers who have ANC visit to be more likely to give birth at health institutions. However, even among women who have ANC follow up, many of them give birth at home, usually without skilled birth attendants. For instance, among women booked for ANC in Ghana, only 38% gave birth at health institutions [8]. Other studies conducted in Nigeria, Senegal and Nepal have shown that institutional delivery rates among women who had at least one ANC visit were 68.5%, 76% and 50%, respectively [9–12]. Studies conducted in northern Ethiopia among women who were booked for ANC have reported the magnitude of home birth to range from 25.3% to 75.3% [13, 14]. The burden of home birth mainly that of unattended delivery is not only limited to maternal health problem, but it also ends up with perinatal and neonatal morbidity and mortality[15].

Various studies conducted in different developing countries and in different part of Ethiopia revealed different determinants of place of birth. Some of these factors are similar for different study areas while some of them are specific for specific study areas. Among the identified factors are lack of access to health facility, educational status of the expectant women, place of residence, increased parity, counseling service obtained during ANC visit, maternal age, age at first pregnancy, age at first marriage, absence of previous obstetric complications, women’s knowledge of pregnancy complications, number of ANC visits, health care providers’ behavior, quality of ANC service and decision maker on place of delivery [13–22].

However, there is very limited evidence regarding the magnitude of home birth and its determinants among ANC-booked women in Ethiopia. Therefore, this Study was designed to assess the magnitude of home birth and its determinants among ANC-booked women in Wolayta Zone of southern Ethiopia.

## Materials and methods

This study was conducted after obtaining ethical clearance from the Institutional Review Board of the College of Medicine and Health Sciences, Hawassa University. All study participants gave a written informed consent to participate in the study. The data obtained from the study participants were handled with strict confidentiality and all responses were deidentified after the second interview was accomplished.

### Study design and setting

A cohort study was conducted among third trimester pregnant mothers who were booked for ANC in public hospitals in Wolayta Zone, southern Ethiopia from February 1 to May 30, 2017. Wolayta Zone is found in the Southern Nations, Nationalities and Peoiples Region of Ethiopia. The zone has one referral hospital, four district hospitals and 70 health centers (5 urban and 65 rural). The report from the Wolayta Zone Health Department indicated that the total number of deliveries reported from the five public hospitals in the year 2015/16 was 7445. Of these, 3511 were from Otona Referral Hospital (100% of the hospital’s annual plan), 1228 from Bonbe Hospital (72.74% of its annual plan), 1142 from Halale Hospital (85.60% of its annual plan), 956 from Bitana Hospital (71.44% of its annual plan), and 608 from Bale Hospital (77.25% of its annual plan).

### Sample size and sampling procedures

The sample size was determined using the sample size calculator of OpenEpi version 2.3 for the two objectives (i.e., for proportion of home birth among ANC-booked women and for determinants of home birth). The sample size for the proportion of home birth among ANC-booked women was determined considering the following assumptions: expected proportion of home birth among ANC-booked women (68%) based on a previous study[18], 95% confidence level, and a margin of error of 4%. Accordingly, the required sample size was 526. Adjusted for an anticipated nonresponse rate of 5%, the required sample size became 554. This is larger than the total sample size calculated for determinants of home birth (level of education, number of ANC visits, respect during ANC and privacy). Hence, 554 was considered to be a sufficient sample size for this study.

Stratified sampling was used to select the required number of study participants. Each public hospital was considered as a stratum. The sample size was then proportionally allocated based on the client flow for ANC, labor and delivery services at each of the five public hospitals in the year preceding the study period. Accordingly, the number of third trimester pregnant mothers recruited in to the study from each hospital was: 261 from Otona Hospital, 92 from Bonbe Hospital, 85 from Halale Hospital, 71 from Bitana Hospital, and 45 from Bale Hospital. Consecutive third trimester pregnant mothers were included into the study until the required number of study participants was fulfilled in each hospital.

### Data collection tools and technique

The data were collected using structured interviewer-administered questionnaire by trained midwives. The questionnaire was initially developed in English by reviewing pertinent literature and translated in to the local Wolaita language and translated back to English to check its consistency.

Data were collected two times. The first data collection was done through a face-to-face interview in the health facility in which the women were having ANC follow up to gather information about basic socio-demographic and obstetric characteristics. Then, their address, name and phone number or the phone number of the Health Extension Worker (HEW) working in their locality were registered. After two weeks of their expected date of delivery, the clients or the HEW working in the respective localities were interviewed through telephone to get information about the place of birth. If the women did not still give birth during the first call, the telephone call was repeated after another two weeks.

The data were collected by five midwives who had basic emergency obstetric training certificate and who were fluent in the local Wolaita language. The data collectors were recruited on a competitive basis from Hawassa University Referral Hospital and from health facilities in Wolaita Zone. The data collectors were assigned for the data collection in a hospital different from the one they are affiliated with.

### Study variables

In this study home birth is the dependant variable while socio-demographic characteristics, obstetric characteristics and mothers’ knowledge were considered as explanatory variables. Women who scored more than the mean score of the knowledge questions on danger signs of pregnancy, and complications during pregnancy and child birth were considered as knowledgeable.

### Data management and analysis

All filled questionnaires were checked for completeness and the data were coded, entered and cleaned using EpiData version 3.1 and exported to SPSS version 22 for analysis. Descriptive analysis was used to describe the study participants by basic background characteristics and to estimate home birth rate among ANC-booked women. Bivariable logistic regression was used to select independent variables for entry into multivariable logistic regression. Variables having p-values less than or equal to 0.25 in the bivariable analysis as well as those considered important based on literature were entered into the multivariable logistic regression model. Then multivariable logistic regression was used to identify the determinants of home birth among ANC-booked women adjusting for possible confounders. Adjusted odds ratios (AORs) with 95% confidence intervals (CIs) obtained from the multivariable logistic regression were used to judge the presence and strength of association between home birth and possible determinants.

## Results

### Socio demographic characters

Initially 554 mothers on ANC were recruited for the study and data on all basic characteristics were collected including their expected date of delivery (EDD). Two weeks after their EDD, clients were re-interviewed for their place of birth through telephone. A total of 505 participants responded to the second interview yielding a response rate of 91.2% for place of birth. Forty one (8.8%) women were lost to follow up for their place of birth after being booked for ANC. The age of the participants ranged from 15 to 38 years with a mean (± standard deviation [SD]) 25.3 (± 4.06) years. Almost all (98.6%) of the participants were married, 409 (73.8%) were protestant, 498 (89.9%) were Wolayta by ethnicity, and 400 (72.2%) had a parity of two or less. The time taken to reach at health facility for ANC service ranged from five minutes to eight hours (see Table 1).

**Table 1:** Socio-demographic characteristics of the study participants, Wolayta Zone public hospitals, May 2017.

Variables	Category	Frequency (N = 554)	Percentage
Age	15–24	202	36.5
	25–29	250	45.1
	30 and above	102	18.4
Marital status	Single	6	1.1
	Married	546	98.6
	Divorced/widowed	2	0.36
Religion	Protestant	409	73.8
	Orthodox	109	19.7
	Adventist	26	4.7
	Muslim	9	1.6
	Catholic	1	0.18
Ethnicity	Wolayta	498	89.9
	Dawuro	22	4
	Others^a^	34	6.1
Residence	Urban	306	55.2
	Rural	248	44.8
Level of education	no formal education	146	26.4
	Primary	183	33
	Secondary and above	225	40.6
Time taken to reach health facility	≤30 minutes	438	79.1
	>30 minutes	116	20.9
Occupation	House wife	332	59.9
	Employed (government	101	18.2
	Private business)	67	12.1
	Other^b^	54	9.7
Access to mass media	No	175	31.6
	Yes	379	68.4
Husband’s education	No formal education	111	20
	Primary school	155	28
	Secondary school	128	23.1
	Above secondary school	160	28.9
Husband’s occupation	Farmer	167	30.1
	Employed	150	27.1
	Private business	181	32.7
	Other^b^	56	10.1

Other ^a^ = Oromo, Amhara, Gamo or Hadiya

Other ^b^ = Students, daily laborer, jobless

### Perceived characteristics of ANC and labour-delivery service, and client’s knowledge about danger signs during pregnancy, labor and delivery

The minimum and maximum gestational ages at first ANC visit ranged from three weeks to 39 weeks with a mean (±SD) gestational age of 19 (± 6.94) weeks. The duration of time they waited for service at ANC clinics ranged from five minutes to eight hours with a mean duration of 43 minutes. More than 95% of the participants reported that they were cared for with respect, got advice on complication of pregnancy, preparedness for birth and explanation about their health condition during ANC visit. Four hundred and sixty two (83.4%) of the participants knew at least half of the eight danger signs of pregnancy and 423 (76.4%) of participants knew at least half of the ten possible complications during labor and delivery (see Table 2).

**Table 2:** Obstetric characteristics and knowledge about obstetric danger signs among ANC-booked women in Wolayta Zone public hospitals, May 2017.

Variables	Category	Frequency (n = 554)	Percentage
Parity	≦2	400	72.2
	>2	154	27.8
Gravidity	≦2	295	53.2
	>2	259	46.8
Number of ANC visit by the time of data collection	One	55	9.9
	Two	101	18.2
	Three	174	31.4
	Four and above	224	40.4
Waiting time for ANC	≦30 minutes	432	77.98
	>30 minutes	122	22.03
Fear to expose genitalia	No	344	62.1
	Yes	210	37.9
Age at first pregnancy	≦18 years	146	26.4
	>18 years	348	62.8
Pregnancy was planned	No	92	16.6
	Yes	462	83.4
Advised on place of birth	No	15	2.7
	Yes	539	97.3
Privacy respected	No	4	0.7
	Yes	550	99.3
Counseled what to expect	No	110	19.9
	Yes	444	80.1
Explained about her health	No	20	3.6
	Yes	534	96.4
Know danger sign of pregnancy	No	92	16.6
	Yes	462	83.4
Know complications of labor and delivery	No	131	23.6
	Yes	423	76.4
Mothers’ perception of who is eligible to give birth in a health institution?	Mothers who have indications	149	26.9
	All mothers	405	73.1
Discussion with husband on place of delivery	No	27	4.9
	Yes	527	95.1
Decision maker on birth place	Me	68	12.3
	Husband	69	12.5
	Together	417	75.2

### Place of birth

Among the 505 participants who responded to the question on place of birth, 437 (86.5%) gave birth at health facilities (either hospital or health center) while 68 (13.5%, 95%CI: 10.5%-16.6%) gave birth at home.

### Determinants of home birth among ANC-booked women

On bivariable logistic regression, maternal age, maternal level of education, parity, residence, gestational age at first ANC visit, time taken to reach at health facility for ANC service, waiting time for ANC service, mother’s knowledge about danger sign of pregnancy and possible complications during labor and delivery, and perception about health facility birth indications were associated with place of delivery. However, on multivariable logistic regression, being an uneducated mother (AOR = 2.46, 95% CI: [1.10–5.10]), late presentation for ANC (>16weeks) (AOR = 2.27, 95%CI: [1.14–4.50]), time taken to reach at health facility for ANC service of greater than 30 minutes (AOR = 8.94, 95% CI: [4.50–17.72]), waiting time of greater than 30 minutes for ANC in health facilities (AOR = 1.18, 95% CI: [1.06–2.30]**)** and lack of knowledge about danger signs of pregnancy (AOR = 4.18, 95%CI: [1.80–9.70]) significantly increased the odds of home birth after being booked for ANC (see Table 3).

**Table 3:** Determinants of home birth among women who were booked for ANC at public hospitals of Wolayta Zone, southern Ethiopia, May 2017.

Variables		Home birth		COR (95%CI)	AOR (95%CI)	p-value
		Yes	No			
Mothers Age	(15–24)	20	170	1	1	
	25–29	29	191	1.3(0.74–2.53)	1.26(0.56–2.82)	
	30 and above	20	75	2.4(1.21–4.76)	1.57(0.57–4.30)	
Mothers level of education	Not educated	24	92	2.52(1.95–8.19)	**2.46(1.10–5.10)**	**0.024**
	Primary school	31	146	3.25(1.64–6.43)	1.29(0.45–3.66)	
	Secondary school and above	20	193	1	1	
Parity	≤2	42	328	1	1	
	>2	26	109	1.86(1.09–3.18)	1.03(0.36–2.90)	
Residence	Urban	22	263	1	1	
	Rural	46	174	3.16(1.84–5.44)	1.18(0.57–2.43)	
Gestational Age at ANC-1	≤16weeks	20	225	1	1	
	>16weeks	48	212	2.55(1.46–4.40)	**2.27(1.14–4.50)**	**0.022**
Time taken to reach at health facility	≤30 minutes	24	375	1	1	
	>30 minutes	44	62	11.09(6.30–19.52)	**8.94(4.50–17.72)**	**< 0.001**
Waiting time for ANC	≤30 minutes	41	342	1	1	
	>30minutes	27	95	2.37(1.39–4.10)	**1.18(1.06–2.30)**	**0.045**
Know danger sign of pregnancy	No	28	61	4.32(2.48–7.51)	**4.18(1.80–9.70)**	**0.001**
	Yes	40	376	1	1	
Know complications of labor and delivery	No	30	99	2.7(1.6–4.57)	1.2(0.54–2.68)	
	Yes	38	338	1	1	
Indications for institutional delivery	All laboring mothers	42	324	1	1	
	Mothers having problems	26	113	1.78(1.04–3.02)	1.25(0.65–2.40)	

Note: COR, crude odds ratio; AOR, adjusted odds ratio; Model: Enter; Hosmer and Lemeshow model fitness: p = 0.60

## Discussion

In this study, the proportion of ANC-booked women who gave birth at home was 13.5% (95% CI: 10.5%-16.6%). This result is consistent with a similar study conducted in Nepal where the rate of home delivery was found to be 15%[19].

On the other hand, this result is lower than the results of other studies conducted in Ghana (68%) [8], Nigeria (31.5%) [9], Senegal (24%) [10, 11], Nepal (50%) [12], Gozamin district of Gojjam, Ethiopia (75.3%) [14], and Debremakos and Fogera districts, Ethiopia (25.3% and 68.4%, respectively) [13, 18]. This is also lower than the proportion of home births stated in the Ethiopian Demographic and Health Survey[1, 5]. The difference of the result of the present study from those of the previous studies can be explained as follows. First, it might be due to the effort made by the Ethiopian government to make all kebeles free of home delivery. Second, it might be due to the difference in study design; in the current study, pregnant women who visited ANC clinics were followed for their place of delivery while the previous studies were retrospective type. The third reason could be due to the gap in time period as some of the above studies were conducted before five years. The difference of the result of the present study from that of EDHS also can be explained as follows. The EDHS covers a wide geographical area with considerable variation in access to health care and health seeking behaviours. It also relies on cross-sectional data which is collected for the five years time preceding the survey date. Besides, it assesses both for ANC follow-up status and place of birth retrospectively. But in the present study, a cohort of pregnant women on ANC were identified from a specific geographical area and followed till birth to ascertain place of birth. Hence, results are for a very recent time period (May 2017) and a specific geographical area and population and outcome ascertainment is prospective. Therefore, in the EDHS the magnitude of home birth is expected to be higher even among those booked for ANC.

The odds of home birth among women who were not educated were 2.46 times higher than the odds of women who attended secondary school and above. This is supported by studies conducted in Nigeria and different parts of Ethiopia [11, 13, 14, 17, 18, 23–27]. This could be due to the fact that educated women have more awareness about the advantage of health facility birth and hence may prefer giving birth in health institutions[1].

Women who waited more than 30 minutes at health facility for ANC had about 1.2 times higher odds of giving birth at home than women who wait less than 30 minutess. If mothers wait long for ANC, then they may feel that they will wait long to get delivery service too. This may discourage them from seeking health facility birth. In line with this, in a study conducted in Nigeria[28], about 29% of mothers who did not give birth in government health facilities had reported long wating time as a cause. Hence, provision of ANC to mothers without much waiting time may serve as a reinforcer for health facility birth.

The odds of home birth among women who started ANC follow up after 16 weeks of gestation were 2.3 times higher than the odds of women who started ANC follow up before 16 weeks of gestation. This is similar with a study conducted in Bahir-Dar, northern Ethiopia [16]. In other studies, this was explained in terms of number of ANC visits. The previous studies have shown that women who have completed four visits were more likely to give birth at health institution [8, 10, 13, 18, 19, 29]. This can be true if the woman starts ANC follow up early in pregnancy as the visit is conducted by time intervals based on focused ANC[2]. Hence, there is high probability for late bookers not to get four visits. In this study, it is difficult to check the effect of number of ANC visit as the data on ANC was collected before delivery; they may or may not have additional ANC visit after the data were collected.

The odds of home birth among women who need more than 30 minutes to reach at health facilities were almost nine times higher than odds of women who need less than 30 minutes to reach at health facilities. This finding is consistent with other studies conducted in northern and southern parts of Ethiopia [18, 25, 30]. In the present study, there were women who reported that they travel eight hours to get to health facility. This can be related to inaccessibility of transportation and inaccessibility of services including delivery service which may discourage giving birth in health facilities.

Furthermore, the odds of home birth among women who lacked knowledge about the danger signs of pregnancy were four times higher than odds of women who were knowledgeable about pregnancy danger signs. This is consistent with studies conducted in northern part of Ethiopia in Alamata, Bahir-Dar and Abergale areas [16, 31, 32]. Women who know about danger signs of pregnancy, possible complications, and prevention methods may prefer health facility birth, while those who have no knowledge of such problem prefer home birth.

This study is not free from limitations. Information about place of birth was obtained from health extension workers for clients who did not have telephone service. This may affect the magnitude of home birth as the health extension workers may under-report the number of home births since they are responsible for mothers’ place of birth in their respective kebeles (small administrative units). Even when the mothers were reached, there may still exist a social desirability bias whereby the mothers are inclined to report giving birth in health facilities rather than at home. Consequently, the magnitude of home birth might have been underestimated.

## Conclusions

In this study, the prevalence of home birth among ANC-booked women is low compared to other studies. Being uneducated, long distance or time taken to reach at a health facility, long waiting time during ANC, lack knowledge about danger sign of pregnancy and late ANC visits were significantly associated with home birth among ANC booked women. It is better if ANC service providers give special attention to women who come from far areas and those who have no formal educations while advising on birth preparedness and pregnancy danger signs. Health programme planners should focus on geographical accessibility of health facility. HEWs, health professionals and all stakeholders working on communication and health education about ANC should give emphasis to frequency and time of ANC. Shortening waiting time during ANC may encourage mothers to visit health facilities for birth.

## Supporting information

S1 File English version questionnaire. (DOCX)

S2 File Wolaytigna version. (DOCX)
