# Supplementary material for: Home birth and its determinants among antenatal care-booked women in public hospitals in Wolayta Zone, southern Ethiopia
Source: PLoS One. 2018 Sep 7;13(9):e0203609. doi: 10.1371/journal.pone.0203609 (PMC6128615; doi:10.1371/journal.pone.0203609)
Supplement: S1 File — (DOCX) [file pone.0203609.s001.docx]

**Annex III: Questionnaire in English**

**Hawassa University, College of Medicine and Health Sciences**

**Part One: Respondent’s socio-demographic characteristics**

| **Sr. No.** | **Questions** | **Alternative choices of response** | **Remark** |
| --- | --- | --- | --- |
|  | Age | __________years |  |
|  | Religion | 1. Ethiopian Orthodox 2. Protestant 3. Muslim 4. Catholic 5. Adventist 6. Other(specify)____________ |  |
|  | Ethnicity | 1. Wolyta 2. Amhara 3. Dawuro 4. Oromo 5. Other(specify)___________ |  |
|  | Marital status | 1. Never married 2. Married 3. Widowed 4. Divorced 5. Separated |  |
|  | Occupation of the mothers | 1. House wife 2. Government employee 3. Nongovernmental employee 4. Private Business 5. Student 6. Others Specify)____________ |  |
|  | Occupation of the Husbands if married | 1. Farmer 2. Government employee 3. Nongovernmental employee 4. Student 5. Private Business 6. Others (Specify)____________ |  |
|  | Residency | - 1. Urban   2. Rural |  |
|  | House Hold Income per month in Birr for urban residency | ____________________ |  |
|  | Amount of cereal income from last year in kuntal (for whom are rural in residency ) | Teff_________________-  Maize________________-  Barley________________-  Potatoes________________-  Wheats________________- |  |
|  | Number of domestic animals (for whom are rural in residency ) | Oxen________________-  Cows________________-  Sheep________________-  Goats________________-  Donkeys________________-  Others________________- |  |
|  | Maternal Educational Level | 1. Don’t educated 2. Read and Write 3. Some of Primary Education 4. Completed Primary Education 5. Some of Secondary Education 6. Completed secondary education 7. Above secondary |  |
|  | Husbands’ Educational Level if married | 1. Don’t educated 2. Read and Write 3. Some of Primary Education 4. Completed Primary Education 5. Some of Secondary Education 6. Completed secondary education 7. Above secondary |  |
|  | How many time it take to reach Health Facility? | __________hr in vehicles  ___________hours on foot |  |
|  | How much is your family size | ___________no. of persons |  |
|  | Do you fear expose your reproductive organ during delivery? | 1. Yes 2. No |  |
|  | Exposure to media in weekly bases (**more than one choice is possible out of three possibilities**) | - 1. Reads a newspaper at least once a week   2. Watches television at least once a week   3. Listens to the radio at least once a week   4. Accesses all three media at least once a week   5. Accesses none of the three media |  |

**Part Two: Obstetrics and Maternal health care factors**

| **Sr. No.** | **Questions** | **Alternative choices of response** | **Remark** |
| --- | --- | --- | --- |
| **201.** | Age at first marriage (at first union ) | In years--------------- |  |
| **202.** | Age at first pregnancy | In years--------------- |  |
| **203.** | Gravidity | ___________no. of pregnancy |  |
| **204** | Parity | ___________no. of parity |  |
| **205** | Expected date of delivery | ____/____/ 2009 |  |
| **206.** | Have you been experienced prolonged labor | 1. Yes 2. No |  |
| **207.** | Where did you give birth your previous last child? | 1. At my own home 2. My parents’ home 3. Health facility 4. TBA home 5. Religious home 6. Other (specify)________ |  |
| **208.** | Experience of bad obstetric history previously | 1. Caesarean section delivery 2. Abortion 3. Still birth 4. IUFD 5. Neonatal loss 6. Others, specify ___________________ 7. No obstetric complication is experienced |  |
| **209.** | Was this pregnancy planned? | 1. Yes 2. No |  |
| **210** | Number ANC visit received? | ______________ in numbers |  |
| **211** | Gestational age at first ANC visit? | ______________ in weeks |  |

**Part three: INSTITUTIONAL CARE RELATED QUESTIONS: Counseling and communication during ANC**

| **Sr. No.** | **Questions** | **Alternative choices of response** | **Remark** |
| --- | --- | --- | --- |
| **301.** | Did the providers explain your health condition with terms that you able to understand? | 1. Yes 2. No |  |
| **302.** | Did the providers explain what to expect during labor and delivery? | 1. Yes 2. No |  |
| **303.** | Did the health providers listen to your questions or concerns? | 1. Yes 2. No |  |
| **304.** | Did the providers respect you? | 1. Yes 2. No |  |
| **305.** | Did the provider protect your privacy during the examinations? | 1. Yes 2. No |  |
| **306.** | How do you rank the behavior of health workers providing ANC services? | 1. Good 2. Fair 3. Bad |  |
| **307.** | How long was the time you spent in waiting to get ANC services? | _________ minute |  |
| **308.** | What do you think the quality of ANC given? | 1. Good 2. Satisfactory 3. Poor |  |
| **309.** | Did you get advice about the need to have delivery at health facilities? | 1. Yes 2. No |  |
| **310.** | When did you get the advice? | 1. During ANC visit 2. During home visit by HEW 3. During contact with TBA |  |

**Part four: Knowledge related questions**

| 401 | Do you Know danger signs during pregnancy and labor? | 1. Yes 2. No |  |
| --- | --- | --- | --- |
| 402 | If “yes” what are they? **** Don’t read the choices, multiple response is possible** | 1. Vaginal bleeding 2. Persistent vomiting 3. Severe headache 4. Blurring of vision 5. Severe upper right upper quadrant abdominal pain 6. Seizure 7. Obstructed labor/ prolonged labor 8. Retained placenta 9. Others, specify ______________________ |  |
| 403 | What are the advantages of pregnancy and delivery related services? | 1. For anticipating problems 2. For early detection of health problems 3. For appropriate management of health problems 4. For better health care to the women 5. For better care to the newborn |  |
| 405 | What complications do you know that can occur during pregnancy? | 1. Vaginal bleeding 2. Severe headache 3. Severe abdominal pain 4. Marked & fast weight gain 5. Amniotic fluid leakage 6. Absence of fetal movement |  |
| 406. | Who are susceptible for pregnancy and delivery complications? | 1. Every mother including myself 2. Primi-gravida-mothers 3. Multi gravid mothers (5 and more) 4. Mothers with multiple pregnancy 5. Mothers with other medical problems |  |
| 407. | What are the complications that can occur during delivery? | 1. Severe hemorrhage 2. Retained placenta (lasting more than 30 minutes) 3. Prolonged labor (lasing more than 12 hours) 4. Loss of consciousness |  |

**Part Five: Decision Related Questions**

| 501 | Do you discuss with your partner about where to deliver? | 1. yes  2. No |  |
| --- | --- | --- | --- |
| 502 | Where is your preference of delivery place? | 1. Home 2. Health facilities 3. Other places, specify-------88 |  |
| 503 | What is your husband’s preference about delivery place? | 1. Home 2. Health facilities 3. Other places, specify--------88 |  |
| 504 | What is your preference about attendant of delivery? | 1. Skilled birth attendant  2. Traditional birth attendant  3. Trained birth attendant  4. Family member or relatives  5. Others, specify---------------88 |  |
| 505 | What is your husband’s preference about attendant of delivery? | 1. SBA  2. TBA  3. Family member or relatives  4. Others, specify-------------88 |  |
| 506 | What is the preference of other family members about place of delivery during? | 1. Home 2. Health facilities 3. Other, specify---------------88 |  |
| 507 | What was the preference of the community about the place of delivery? | 1. Home 2. Health facilities 3. Other, specify----------------88 |  |
| 508 | Who will make decision about your delivery place? | 1. Myself  2. My husband  3. Both me & my husband  4. Others, specify--------------88 |  |
| 509 | Who decides on the cost related to health care/ for referral or reaching health facility? | 1. Myself  2. My husband  3. Both me & my husband  4. Others, specify--------------88 |  |

**Part six: Ideas on institutional delivery and reasons given to Place of delivery**

| **Sr. No.** | **Questions** | **Alternative choices of response** | **Remark** |
| --- | --- | --- | --- |
| **601** | What do you think about necessity of institutional delivery? | 1. It is necessary 2. Not necessary |  |
| **602** | What do your families think about necessity of institutional delivery? | 1. They all think it is Necessary 2. Father did not think necessary 3. Family did not think necessary 4. Husband did not think necessary 5. Not customary |  |
| **603** | Whom do you prefer to attend your delivery among skilled health care provider and TBA? | 1. Health care provider 2. TBA |  |
| **604.** | Where will you delivery your current pregnancy? | 1. Home 2. My own home 3. My parent’s home 4. My relatives home 5. Others, specify 6. Health facility 7. Public Hospital 8. Private Clinic 9. NGO’s Clinic 10. Health center 11. Health post 12. Others, specify _ | **If ‘b’ Skip to Q.**607 |
| **605.** | If your answer is “a” for the above question, why do you prefer home delivery? **Multiple answer is possible** | 1. Institutional delivery is not necessary 2. Providing home delivery is our culture 3. Institutional delivery is too costly 4. Health professionals do not allow family members to accompany in labor ward 5. Dislike behaviors of health workers at health institution 6. Too far health facility and lack of transport 7. Poor quality of service in health institution 8. Labor was simple and normal 9. Wishes to deliver at home where relatives are nearby 10. Husband/ family don’t allow 11. Others, specify________________ |  |
| **606.** | Who will assist you while delivery? | 1. TBA 2. Relatives 3. No one 4. Others, specify______________________ |  |
| **607.** | If your answer is “b”, why did you want to deliver your baby in that particular place? **Multiple answer is possible** | 1. Close to where I live 2. High quality services 3. Good approach of health workers 4. Little expenses to deliver in this particular institution 5. Because I was told to have institutional delivery during ANC 6. Because I had previous caesarian section 7. I had previous difficult labor 8. Because I fear complications 9. Others, specify________________ |  |

# 
