# Supplementary material for: Home birth and its determinants among antenatal care-booked women in public hospitals in Wolayta Zone, southern Ethiopia
Source: PLoS One. 2018 Sep 7;13(9):e0203609. doi: 10.1371/journal.pone.0203609 (PMC6128615; doi:10.1371/journal.pone.0203609)
Supplement: S2 File — (DOCX) [file pone.0203609.s002.docx]

# **Annex IV: Wolaytigna Version**

1. **WOLAYITTETTO OYISHSHA**

**Hawaasa univurshshiyaan aakimenne payyatteatta saynnisiyaan Kollogiyaan**

**Qaalaa maachchaa**

Bonchcho oyishshettiyageeto;

Aymmalee? Taani ______ geettettayiis. Ta Hawaasa Univurshshiyaan aakimenne payyatteatta saynnisiyaan kolojiyian oottayiis. Ha’i ta hagaan Hawaasa Univurshshiyaan Wolayitta zooniya maaretappe kase kaaluwa kawo payyattetta nagiiya keetan kalli simiddi aappun shaara maccaasatti soon yeliyaakkonne ayiis yeliyaakkoo qoncciis eranaawu pilggiyagaas maaddiya oyshshaa oychchayidda de’ayiis.

Ha pilggettawu waanna halchchoy daro machchaasti ayiis sooni yeliyaakoo payyattattaa naagiya asata, kawo oso keetta, kawonne buzo payyattetta naagiyaa keettattanne wako oso keetta gidenna dirjjitetuus qonccisanaasa. Hegaa gishshaawu ha pilggettawu maaddiya oyshshata nenna oychchanna koyaas. Ne imiyoo zaaroti shaara maccaasati ayiis sooni yeliyo dumma dumma gaasota eranawu keehippe maddoosonna. Neeni ha oyshsha doomada woyikko muleera agganna giikko he ne maata. Gidoppe attin neeni tawu odiyoobay aybbika xuuran gam’’anaagaadaan mule qaala gellayidda ne ha ekkanna koshshiyo haggazuwappe aybbikka attenaagaa geeshsha qonchchisayiis.

Oyshshaa zaaranawu eeno gaasakka?

1. Eenno giikko , doomma
2. Chii giikko essadda oyshshetiyagaa galata

Oyshshaa koode -----------------------------------

De’iyoosay: woradaa_______________ kabaliyaa_________________ heera

Silkke paydduwa 1. ---------------------

2.________________

Oychchiyaaga suntta__________________________ paramaa _____ gallassa______

**Waanna pilggiyagee: Melese S. silkkee payddoy : +251 912 78 05 77/ 0921799284**

**Annex III: oyshshaa**

**Menttuwa 1: oyshshettiyaageetu kane hantata**

| **Sr. No.** | **Oyshshatta** | **Imettida doorota** | **yigetta** |
| --- | --- | --- | --- |
|  | Layittaay aappune | __________layitta |  |
|  | Ammanooy aybbee? | 1. Tooppe orttodooqise 2. pirosttantte 3. isilaama 4. kaatolike 5. adiventtistte 6. haray diikoo (qonccisa)__________ |  |
|  | Sheeshshaay ayibee? | 1. Wolayitta 2. Amaara 3. Dawuro 4. Orommo 5. haray diikoo (qonccisa)__________ |  |
|  | Gelluwaa hanottay | 1. mule gelabeeyikke 2. gelaas 3. keettaway hayiqqiis 4. sawo/yeddooro 5. dumma deettees |  |
|  | Ne oosooy aybbee? | 1. So asa 2. Kawo oosanchcha 3. Kawo gidenna dirjjite oosanchcha 4. Gille oosanchcha 5. tamaare 6. haray diikoo (qonccisa)__________ |  |
|  | Keettawaa oosooy? | 1. gashshanchcha 2. Kawo oosanchcha 3. Kawo gidenna dirjjite oosanchcha 4. Gille oosanchcha 5. tamaare 6. haray diikoo (qonccisa)__________ |  |
|  | De’ooy awaee? | - 1. katamaa   2. gaxariyaa |  |
|  | Intte soo aginnaa geliyaa miishsha likkee | ____________________ |  |
|  | Layttaan intte soo geliya kattay kunttaliyaan (gaxariyaa asas ) | gaashshe_________________-  badalay________________-  banggay________________-  donnoy________________-  Wheats________________- |  |
|  | Intte soon de’iya mehee (gaxariyaa asas) | booray________________-  miizay________________-  dorssay________________-  deeshshay________________-  haree________________-  haray diikko________________- |  |
|  | Aappuntta gakkanaw taamaaraddi? | 1. Mule taamaarabeykke 2. Nabbabuwaanne xaapuwaa danddayayiis 3. Doometa taamaaraas 4. Koyiro xekka wurssaas 5. Naa’antto xekkapee bagaas 6. Naa’antto xekka wurssaas 7. Naa’antto xekkappe bolla |  |
|  | Nekeettaway appuntta gakkidee? | 1. Mule taamaarabeynna 2. Nabbabuwaanne xaapuwaa danddayees 3. Doometa taamaariis 4. Koyiro xekka wurssiis 5. Naa’antto xekkapee bagiis 6. Naa’antto xekka wurssiis 7. Naa’antto xekkappe bolla |  |
|  | Ne sooppe matan de’iya payyattetta noagiyoo keetta gakkanawu aykkeesa wodiya ekkii? | toggiyoobaan __________ saatiiya  tohuwaan ___________ saatiiya |  |
|  | Muleeraa soon de’yaa asaa payidooy | ___________ |  |
|  | Maareta woode ne maccattetta bolla asi be’iiyode yayyay? | 1. Ee yayyaas 2. Chii yayyikkee |  |
|  | Guuxiis giishshin Saamintta garssaan ooratta oduuwa awuppee siyaay (**issuwaappe dariya zaarooy danddayettees**) | - 1. saaminttan issitto Gaazeexaa nababayiis   2. saaminttan issitto Telebizhzhiiniyaa be’ayiis   3. saaminttan issitto Radooniyaa ezigaatiis   4. heezaakka saaminttappe demmayiis   5. aybbakka demma errikke |  |

**Menttuuwa 2: shaaranne maccaasaa payyattettaa xeeliyaagan**

| **Sr. No.** | **Oyishshaa** | **Zaaruwaa** | **Yigetta** |
| --- | --- | --- | --- |
| **201.** | Koyiroo azinaa aappuuntta layittan gelaaddi? | _________ layittaan |  |
| **202.** | Koyiroo shaaraa aappuuntta layittan shaaraddi? | _________ layittaan |  |
| **203.** | Muleera aappuutto shaaraddi? | ___________tto |  |
| **204** | Muleera aappuutto yeladdi? | ___________ tto |  |
| **205** | Ha’ii awudde yelayiis gaada qoppayyi? | ____/____/ 2009 |  |
| **206.** | Neeyo maaretay gam’’ii erri? | 1. Ee gam’’ees 2. Chii gam’’enna |  |
| **207.** | Wurssetta na’aa awun yeladdi?? | 1. Ta soon 2. Nu soon 3. Payyattetta naagiya keetan 4. Hiilla soon 5. Woossiyo keettan 6. Haray diikko qonccissa______ |  |
| **208.** | Kase shaaratuun metidab aybbe | 1. Oppiraasoniyaan yelooga 2. boshshaa 3. yeletiidi na’ay hayqqogaa 4. uluwan na’ay hayqqogaa 5. gaccinuwan na’ay hayqqooga 6. Haray diikko qonccissa______ 7. Aybba metooyikka bawa |  |
| **209.** | Ha shaara halchchada shaaraddi? | 1. Ee 2. gidenna |  |
| **210** | Yeluwappe kase kaaluwa apputto kaalladdi? | ______________ tto |  |
| **211** | Koyiroo kaaluwawu appuntta aginan yaaddi? | ______________ tta |  |

**Menttuwa 3: payyattetta naagiya keetta kaaluwa xeeliyagan : marettappe kase kaaluwa zoriyanne maqquwa baggara**

| **Sr. No.** | **Oyishshaa** | **Zaaruwaa** | **Yigetta** |
| --- | --- | --- | --- |
| **301.** | Ne shaaraa payyattetta kaaliyageetti ne payyattetta hanotaa neyyo gelanaadan qonccisiyonna? | 1. Ee 2. gidenna |  |
| **302.** | Ne shaaraa payyattetta kaaliyageetti maareta woode aybbi aybbi hananaako qonccisidona? | 1. Ee 2. gidenna |  |
| **303.** | Ne shaaraa payyattetta kaaliyageetti neen oychchiyoba woyikko odiyooba ezigiddi siyoona? | 1. Ee 2. gidenna |  |
| **304.** | Ne shaaraa payyattetta kaaliyageetti nena bonchchiyoona? | 1. Ee 2. gidenna |  |
| **305.** | Ne shaaraa payyattetta kaaliyageetti ne gatiya naagiyoona? | 1. Ee 2. gidenna |  |
| **306.** | Ne shaaraa payyattetta kaaliyageetu eeshshaa aybbidan xeellay? | 1. Keehippe lo’o 2. Lo’o 3. Likayinne 4. Iita |  |
| **307.** | Shaaraa Kaaluwawu naagishin aykeesa woode wurri? | _________ daqiiqaa |  |
| **308.** | Shaaraa Kaaluwaa sanaccattetta aybbidan xeeliay? | 1. Lo’o 2. Likayinne 3. Iita |  |
| **309.** | Payyattetta naagiyo keettan yelana mala zoriya ekka eray? | 1. Yes 2. No |  |
| **310.** | Awude awude ekkaddi? | 1. Shaaraa Kaaluwaa wode 2. Soon heera payyattetta naagiyageettuppe 3. hiillanchchattuppe |  |

**Menttuwa 4: akeekaa xeelliyagaan**

| 401 | Shaaraanne maareta wode beettiya iita malaatata eray? | 1. Ee 2. gidenna |  |
| --- | --- | --- | --- |
| 402 | Ee giikkoo eti aybbe aybbe? **** zaarota odoppa, issuwappe daruwa dooranaawu danddayettees** | 1. suutaa 2. aggenna cooshshaa 3. wolqqaama huphe zawuriya 4. yiiccuwaa 5. ushshachcha bagga tiara sahuwaa 6. kokorssiyogaa 7. maarettay gam’iyogaa woyikko yeletenan ixxiyogaa 8. geedobay wodhdhenagaa 9. haray diikko qonccisa___________ |  |
| 403 | Shaaranne maareta kaaluwa goo’ati aybbe aybbe? | 1. Metuwa kasetiddi hirgganawu 2. Metoy diikko kasetiddi eranawu 3. De’iya metotta maaran xaletanwu 4. Aayetu payyattetta naaganawu 5. Qeeri na’’tu payyattetta naaganawu |  |
| 405 | Shaaraa wode gakkiya metottuppe aybba aybba eray? | 1. suuttaa 2. wolqqaama huuphe zawuriya 3. wolqqaama ulo sahuuwa 4. eesuuwan erettiya orddettetta 5. kuukulssay xokkiiyogaa 6. na’ay uluwan qaaxxennaagaa |  |
| 406. | Shaaraanne maaretta wode dariin waayettees ne giiyoy oonee? | 1. ubba maccasa tanaara gattin 2. koyiroo yeliyaa maccaasaa 3. 5ppe boollaa yela maccaasa ) 4. Menttiya shaara maccaasa 5. Hara hara payyattetta metooy de’iyo maccaasaa |  |
| 407. | Maaretta wode gakkiya metottuppe aybba aybba eray? | 1. Wolqqaama suuttay gukiyoogaa 2. Geedobay gam’iiyoogaa (30 daqqiqappe bollaa) 3. Maaretaay gam’’iyooga (12 saatiyaappe bollaa) 4. Kaha dogiyoogaa |  |

**Mettuwa 5: yoohuwa qashshuwaba xeeliyaagan**

| 501 | Awuun yelanaakko nekeettawaara zorettay? | | 1. Ee 2. gidenna | | |  | | |
| --- | --- | --- | --- | --- | --- | --- | --- | --- |
| 502 | Yeluwawu neen dooriyosay awee? | 1. soona 2. payyattetta naagiyo keettana 3. harasay diikko, qonccisa-------88 | |  | | |  |  |
| 503 | Nekeettaaway neeni awun yeliyaakoo dosii? | 1. soona 2. payyattetta naagiyo keettana 3. harasay diikko, qonccisa-------88 | | |  | | |  |
| 504 | Nna oonii yelisiyaakoo dossay? | 1. tamaarida eranchchay  2. meeze hilaanchcay  3. loohida hilaanchchay  4. so asaappe woyikko dabboy  5. harasay diikko, qonccisa-------88 | | |  | | |  |
| 505 | Nekeettawayshshin ? | 1. tamaarida eranchchay  2. meeze hilaanchcay  3. loohida hilaanchchay  4. so asaappe woyikko dabboy  5. harasay diikko, qonccisa-------88 | | |  | | |  |
| 506 | Intte soo assay neeni awun yeliyakko dosiyonaa? | 1. soona 2. payyattetta naagiyo keettana 3. harasay diikko, qonccisa-------88 | | |  | | |  |
| 507 | Inttte heera assay darooto awun yeliyooga dosiyoonaa? | 1. soona 2. payyattetta naagiyo keettana 3. harasay diikko, qonccisa-------88 | | |  | | |  |
| 508 | Ne yeliyo sohuuwa qoppa ooni qaccii? | 1. neeni  2. ne keettaway  3. naa’ay  4. harasay diikko, qonccisa-------88 | | |  | | |  |
| 509 | Payyattetta naaguwawu qanxxiyyo biraabanne hara payyattetta keettawu shiriyyo/moyisiyo hanotta qoppa ooni qaccii? | 1 neeni  2. ne keettaway  3. naa’ay  4. harasay diikko, qonccisa-------88 | | |  | | |  |

**Menttuwa 6: payyattetta naagiyo keetaan yeliyogaanne yeliyo sohuuwa dooruwa xeeliyaagan**

| **Sr. No.** | **Oyishshaa** | **Zaaruwaa** | **Yigetta** |
| --- | --- | --- | --- |
| **601** | Payyattetta naagiyoo keettaan yeliyogee koshshii koshshenne ? | 1. Ee koshshees 2. Chii koshshenna |  |
| **602** | Intte soo assay Payyattetta naagiyoo keettaan yeliyogaa koshshees giid qoppiyoona? | 1. Ee ubbaykka qoppoosona 2. Taaway dariin qoppenna 3. Chii oonnekka qoppenna 4. Takeettaaway qoppenna 5. Mule dariin erettenna |  |
| **603** | Taamaariyan loohida payyattettaa eranchchaappenne loohida meeze hillanchchappe ooni nena maretisanna mala koyay? | 1. Taamaariyan loohida payyattettaa eranchchaa 2. Meeziyaan loohida hillanchcha |  |
| **604.** | Ha shaarra awuun yelanawu qoppay? | 1. Soon 2. Ta soon 3. Taawaa soon 4. Dabbuwa soon 5. Woosa keettan 6. harasay diikko, qonccisa-------88 7. Payyattetta naagiyoo keettan 8. Kawo hosppitaliyan 9. Gille kilinkkiyan 10. Kawoba giddenna kilinkkiyan 11. Payyattetta naagiyo xaabiyan 12. Xeena keellan 13. harasay diikko, qonccisa-------88 | **If ‘b’ Skip to Q.**607 |
| **605.** | Ne zaaroy ‘a’ gidikko ayissi payyattetta naagiyo kettan doorabeykki? **Chora dooroy danddayetees** | 1. payyattetta naagiiyo kettaan yeliyoogee maaddenna 2. soon yeliyooge woga giddiyo gishsha 3. payyattetta naagiiyo kettaan yeliyoogee al’’o miishsha oychchiyoo gishsha 4. payyattettaa naagiyoo keettan so assay gelenna gishsha 5. payyattetta naagiya asaa eeshsha taan dosenna gishsha 6. payyattetta naagiiyo kettaay keehippe haakkiyo gishsha 7. payyattetta naagiiyo kettaan demmiyo hagaazoy lo’enna gishsha 8. tana maaretay waayessenna gishsha 9. tadabboy zaree diyosaan yelanawu koyyiyo gishsha 10. takeettaway/ nusoo assay yeddenna gishsha 11. harasay diikko, qonccisa-------88 |  |
| **606.** | Neen yeliyoo wode ooni nena maadiyaakko lo’oo? | 1. Loohidi maaretisiyaageeti 2. Dabboy 3. Oone 4. harasay diikko, qonccisa-------88 |  |
| **607.** | Ne zaaroy “b”, gidikko ayssi hesohuuwan yelana koyyaddi? **Chora dooroy danddayetees** | 1. takeetawu mattattiyo gishsha 2. keehippe nashshetiya hagaazuwa immiyo gishsha 3. yelisiyaageeti asaa keehippe bonchchiyo gishsha 4. daro miishshaa oychchenna gishsha 5. maarettappe kasa kaaluwa wode yaan yelanadaan oddetto gishsha 6. kase opiraasooniyan yelo gishsha 7. kase maaretay gam’oo gishsha 8. ixi iitid yiya maareta metuwa yayyo gishsha 9. harasay diikko, qonccisa------- |  |
